# HBcAb positivity increases the risk of postoperative complications after extended hemihepatectomy for hilar cholangiocarcinoma

**DOI:** 10.1002/cam4.5740

**Published:** 2023-02-27

**Authors:** Wen‐Qiang Wang, Guang‐Yuan Xu, Jian Li, Bin‐Yong Liang, Jiang Li, Mei‐Long Lin, Xiao‐Ping Chen, Er‐Lei Zhang, Zhi‐Yong Huang

**Affiliations:** ^1^ Hepatic Surgery Center, Tongji Hospital, Tongji Medical College Huazhong University of Science and Technology Wuhan China

**Keywords:** HBsAg negative, hemihepatectomy, hepatitis B core antibody, hilar cholangiocarcinoma, postoperative complications, surgical outcomes

## Abstract

**Background:**

Hepatitis B core antibody (HBcAb) positivity is considered a prior hepatitis B virus (HBV) infection. However, little is known about the effect of HBcAb positivity on surgical safety for hilar cholangiocarcinoma (hCCA). The present study aims to investigate the role of HBcAb positivity on postoperative complications of hCCA.

**Methods:**

A retrospective analysis was performed on the status of HBcAb positivity, liver fibrosis, perioperative surgical complications, and long‐term outcomes of hCCA patients with Hepatitis B surface antigen (HBsAg) negativity who underwent surgical treatment in Tongji Hospital from April 2012 to September 2019.

**Results:**

HBcAb positivity with negative HBsAg occurs in 137 hCCA patients (63.1%). A total of 99 hCCA patients with negative HBsAg underwent extended hemihepatectomy, of whom 69 (69.7%) and 30 (30.3%) were HBcAb‐positive and HBcAb‐negative, respectively. Significant fibrosis was detected in 63.8% of the patients with HBcAb‐positive, which was markedly higher than those with HBcAb‐negative (36.7%) (*p* = 0.016). The postoperative complications and 90‐day mortality rates were 37.4% (37/99) and 8.1% (8/99), respectively. The incidence of postoperative complications in HBcAb‐positive patients (44.9%) was significantly higher than that in HBcAb‐negative patients (20.0%) (*p* = 0.018). All the patients who died within 30‐day after surgery were HBcAb‐positive. Multivariate analysis showed that the independent risk factors for complications were HBcAb positivity, preoperative cholangitis, portal occlusion >15 min, and significant fibrosis. There were no significant differences in recurrence‐free survival (RFS) and overall survival (OS) between HBcAb‐positive and HBcAb‐negative patients (*p* = 0.642 and *p* = 0.400, respectively).

**Conclusions:**

HBcAb positivity is a common phenomenon in hCCA patients from China, a country with highly prevalent HBcAb positivity. The status of HBcAb‐positive markedly increases the incidence of postoperative complications after extended hemihepatectomy for hCCA patients.

## Abstract 2

The present study aims to evaluate the prevalence of HBcAb positivity in hCCA patients and explore the effect of HBcAb positivity on postoperative complications of hCCA patients after curative resection. Among all the 217 patients, 160 (73.7%) patients were HBcAb positive. Data of 99 HBsAg negative patients who underwent curative resection were analyzed, and the HBcAb positivity markedly increased the risk of postoperative complications and mortality after curative resection for hCCA. Surgeons should pay more attention to HBcAb status in hCCA patients with negative HBsAg, which could help them to select optimal surgical modalities.

## 1 INTRODUCTION

Cholangiocarcinoma (CCA) can be classified into intrahepatic CCA (iCCA), hilar CCA (hCCA), and distal CCA (dCCA). Among them, hCCA is an epithelial carcinoma, also known as Klatskin tumor, which likely originates from the biliary tree and arises between the secondary bile duct and the cystic duct, accounting for approximately 50%–75% of CCAs.^1^ hCCA is closely associated with primary sclerosing cholangitis, biliary cysts, hepatolithiasis, and chronic liver disease.^2^, ^3^ Surgery is the most effective treatment for hCCA patients.^1^, ^4^ Due to the peculiar anatomical location and predisposition of hCCA to vascular invasion, hemihepatectomy combined with caudate lobectomy is considered as the standard surgical strategy in many medical centers to increase tumor resectability and improve long‐term outcomes.^5^, ^6^, ^7^, ^8^ However, the incidence of postoperative complications and the mortality associated with such surgical resection are relatively noticeable.^7^, ^9^

Hepatitis B core antibody (HBcAb) positivity is an abnormal index that appears after infection with hepatitis B virus (HBV); it typically exists in the serum for a long‐time; thus, it is regarded as a sign of a past infection.^10^, ^11^ Hepatitis B surface antigen (HBsAg) is the hallmark of infection, and it is positive in the early stage of acute infection and persistently in chronic infection.^11^ HBcAb positivity with negative HBsAg is also called prior HBV infection.^10^, ^12^ China is one of the susceptible countries to HBV infection, and the overall prevalence of HBsAg positivity and HBcAb positivity is still remarkable (up to 7.2% and 43.2%, respectively).^13^, ^14^ Previous studies showed that HBV was one of the risk factors for iCCA, and HBsAg positivity will significantly increase the incidence of complications after liver resection.^15^, ^16^ However, the incidence of HBcAb positivity in hCCA patients and the impact of HBcAb positivity on the safety of hCCA patients who received surgery have never been reported. Therefore, the present study aims to evaluate the prevalence of HBcAb positivity in hCCA patients and explore the effect of HBcAb positivity on postoperative complications of hCCA patients after curative resection.

## 2 PATIENTS AND METHODS

### 2.1 Patients' selection

A retrospective study was conducted on consecutive hCCA patients who underwent surgery in the Department of Hepatobiliary and Pancreatic Surgery, Tongji Hospital, Tongji Medical College, Huazhong University of Science and Technology from April 2012 to September 2019.

**FIGURE 1:**
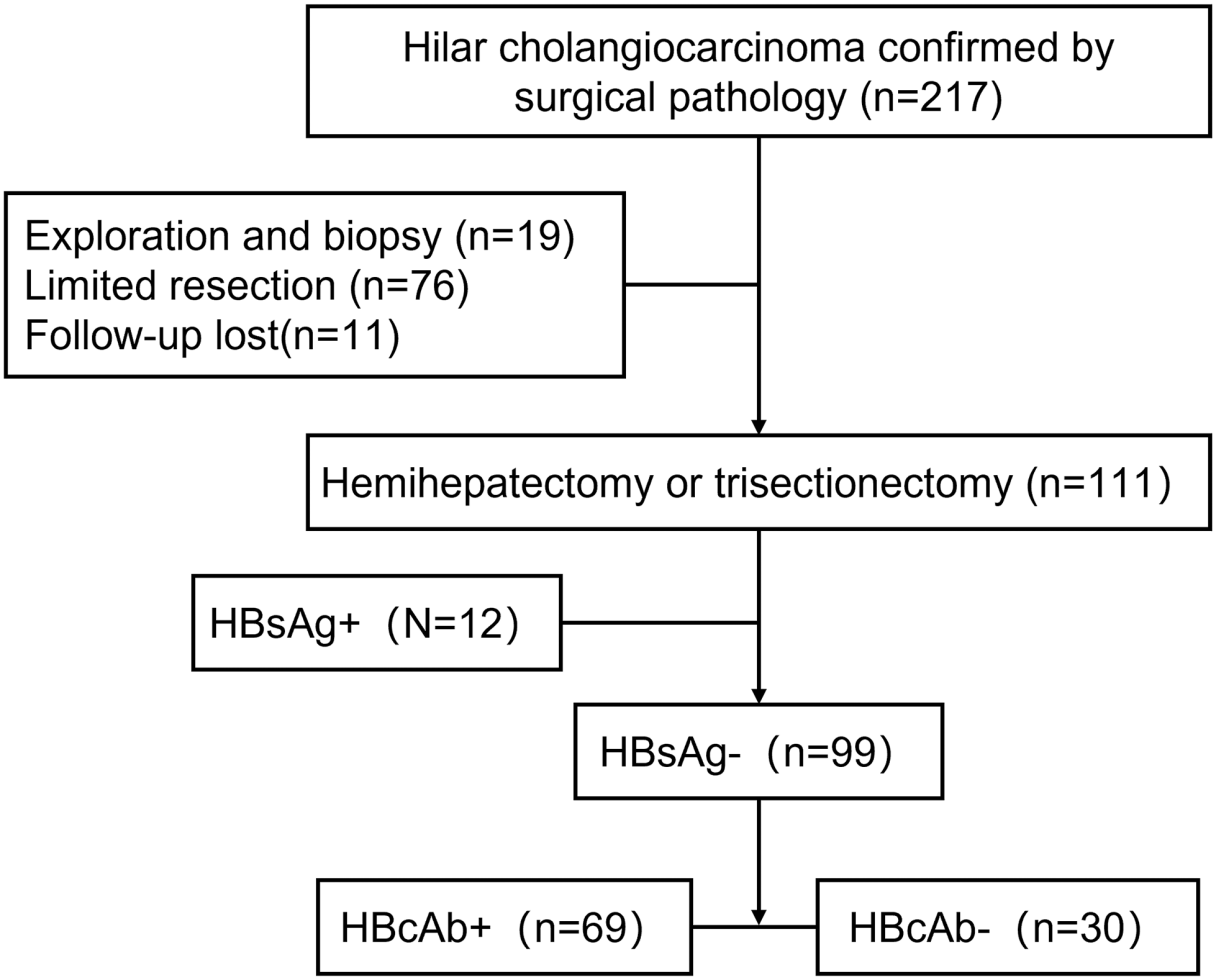
The flowchart of the patient selection.

The inclusion and exclusion criteria were as followings. Inclusion criteria: (1) Patients were pathologically diagnosed with hCCA; (2) Underwent extended hepatectomy (hemihepatectomy combined with caudate lobectomy); (3) Availability of complete clinicopathological data. Exclusion criteria: (1) Underwent exploration and biopsy; (2) Underwent limited resection; (3) Patients had other malignant tumors or severe systemic dysfunction. Figure 1 shows the patient selection flowchart for the study.

### 2.2 Data collection

Patients' demographic, clinical data, and the existence of postoperative complications were collected and analyzed. HBV serological markers were determined by the chemiluminescent microparticle immunoassay method (Architect‐i2000 SR, Abbott, Dublin, Ireland), in which HBsAg ≥0.05 IU/mL, hepatitis B surface antibody (HBsAb) ≥10 IU/mL, hepatitis B e antigen (HBeAg) ≥1 S/CO, hepatitis B e antibody (HBeAb) ≤1 S/CO, and HBcAb ≥1 S/CO indicate the positive result. The patients were diagnosed with cholangitis when they showed fever, jaundice, and abdominal pain (Charcots triad). According to the Bismuth‐Corlette classification of hCCA,^17^ the tumor was classified as type I, II, IIIa, IIIb, and IV. Liver fibrosis was determined by Masson's Trichrome staining of the formalin‐fixed paraffin‐embedded samples. According to the Laennec staging system, liver fibrosis or cirrhosis was categorized into five stages: F0 (no definite fibrosis), F1 (minimal fibrosis), F2 (mild fibrosis), F3 (moderate fibrosis), and F4 (cirrhosis).^18^ Significant fibrosis was defined as ≥F2 and advanced fibrosis as ≥F3.

Postoperative complications and criteria were defined as follows: postoperative pleural effusion refers to the B‐ultrasound exploration in sitting and standing positions and the depth of pleural effusion to be more than 3 cm, or a pleural fluid that requires a thorax puncture or drainage; massive ascites refers to postoperative daily ascitic fluid drainage from abdominal drains exceeding 10 mL/kg of preoperative body weight, at least 3 days, or ascites requiring an invasive procedure comprising percutaneous puncture or drainage (radiology or surgery).^19^, ^20^, ^21^ Postoperative infection refers to signs of inflammation and blood or drainage fluid cultures positive for pathogenic bacteria.^22^ According to the criteria presented by the International Study Group of Liver Surgery, postoperative bile leakage was defined as the drainage of intra‐abdominal fluid with an increased bilirubin concentration (at least three times the serum bilirubin concentration) on or after postoperative Day 3.^23^ Posthepatectomy liver failure (PHLF) was defined as an increasing international normalized ratio (INR; or decreasing prothrombin time) and elevating serum bilirubin concentration on or after postoperative Day 5 (compared with the values of the previous day).^24^ In the present study, we only counted the occurrence of grade B/C PHLF.

### 2.3 Preoperative assessment and surgical procedure

Preoperative liver function and residual liver volume were considered comprehensively, and timely adjustment and treatment of preoperative abnormalities. In the case of obstructive jaundice and cholangitis, biliary drainage was performed in the remnant liver until the total bilirubin concentration decreased by more than half. Percutaneous transhepatic cholangial drainage (PTCD) was used in most cases, while some patients underwent endoscopic biliary drainage (EBD). Assessment of liver function was consistent with the indocyanine green test (ICG) performed after bile drainage reduced serum total bilirubin concentration; we set the safe limit for the ICG retention value as <10% at 15 min for liver resection. In these patients, at least 30% of the functional liver volume (estimated by a preoperative three‐dimensional CT scan) could be preserved after hemihepatectomy or trisectionectomy. Patients with a high level of HBV DNA were required to receive effective antiviral therapy and have a viral load of less than 100 IU/mL at screening. Standardize surgical procedures to minimize surgical risks. All patients underwent left or right hemihepatectomy or trisectionectomy combined with caudate lobectomy, extrahepatic biliary resection, and local lymphadenectomy. Hemihepatic vascular inflow occlusion or the intermittent Pringle maneuver was used to minimize blood loss during parenchymal resection.

### 2.4 Statistical analysis

Statistical analysis and graphical illustration were undertaken using the software SSPS 24.0 (IBM Corp., Armonk, NY, USA) and GraphPad Prism 8.0 (GraphPad Software Inc., San Diego, CA, USA). Continuous variables with normal distribution were presented as mean (standard deviation [SD]), and those with abnormal distribution were reported as median (interquartile range [IQR]). As appropriate, the frequencies of categorical variables were compared using the Pearson χ^2^ or Fisher's exact test. The independent‐samples *t*‐test compared mean values of continuous variables (normally distributed), and the Kaplan–Meier (K‐M) method was used to estimate overall survival (OS). Uni‐ and multivariable analyses of risk factors for complications were performed using logistic regression, univariate comparisons of survival and multivariate analysis were performed using the Cox proportional hazards model, and all variables with a *p*‐value of 0.10 or lower at univariable analyses were entered into the multivariable analyses with backward selection. Multivariable logistic model was fitted using backward stepwise regression. *p* < 0.05 was considered statistically significant.

## 3 RESULTS

### 3.1 Patients' demographic and HBV infection status

A total of 217 consecutive hCCA patients were retrospectively studied. Among these patients, 99 patients with HBsAg negativity who underwent curative resection were enrolled in this study and divided into the HBcAb+ (*n* = 69) group and HBcAb‐ (*n* = 30) group according to the status of HBV infection. Figure 1 shows the flowchart of the patient selection. Of the 217 patients who underwent surgery, 160 (73.7%) patients were HBcAb+, 23 (10.6%) cases were HBsAg+, 3 (1.4%) cases were HBeAg+, and 73 (33.6%) cases were HBeAb+; Meanwhile, we found that all patients with HBsAg+ had HBcAb+, HBcAb+ with HBsAg‐ occurs in 137 hCCA patients (63.1%). Besides, 57 (26.3%) cases had HBV‐negative markers (including all negative or only HBsAb+). There were only two patients with hepatitis C, and neither of them underwent extended hemihepatectomy.

### 3.2 Clinical data and liver fibrosis, postoperative mortality, and complications

Data of 99 HBsAg‐negative patients who underwent extended hemihepatectomy were analyzed, in which the incidence of postoperative complications and 90‐day mortality were 33.3% (33/99) and 8.1% (8/99), respectively. There were 69 (69.7%) and 30 (30.3%) patients in HBcAb+ and HBcAb‐ groups, respectively. The surgical procedures and postoperative complications of the two groups are shown in Table 1 (some patients had simultaneously two or more complications).

**TABLE 1:** Clinical characteristics of patients (*n* = 99).

	HBcAb+(*N* = 69)	HBcAb‐(*N* = 30)	*p* value
Sex, male, *n* (%)	41 (59.4)	18 (60.0)	0.957
Age, years	57 (50–62)	58 (55–64)	0.145
>60, *n* (%)	23 (33.3)	13 (43.3)	0.342
Jaundice, *n* (%)	49 (71.0)	25 (83.3)	0.195
Caroli disease, *n* (%)	8 (11.6)	2 (6.7)	0.719
Primary sclerosing cholangitis, *n* (%)	9 (13.0)	2 (6.7)	0.496
Choledochal cysts, *n* (%)	5 (7.3)	3 (10.0)	0.695
Hepatolithiasis, *n* (%)	10 (14.5)	5 (16.7)	0.768
Alcohol drinking, *n* (%)	24 (34.8)	12 (40.0)	0.654
Preoperative cholangitis, *n* (%)	17 (24.6)	9 (30.0)	0.577
Preoperative biliary drainage, *n* (%)			0.352
PTCD	43 (63.8)	23 (50.0)	
EBD	3 (7.2)	2 (6.7)	
None	23 (29.0)	6 (43.3)	
TB, μmol/L	52 (35–106)	47 (32–90)	0.885
Hb, g/L	128 (113–139)	129 (123–148)	0.209
PT, INR	1.04 (1.00–1.09)	1.07 (1.01–1.13)	0.084
ALT, U/L	54 (36–72)	58 (42–70)	0.948
Alb, g/L	37 (36–39)	38 (35–40)	0.514
CEA, ng/mL	5.2 (3.9–6.3)	5.1 (4.1–6.2)	0.860
CA19‐9, U/mL	417 (51–1977)	343 (136–1252)	0.711
*Operative and pathologic data*			
Bismuth‐Corlette type, *n* (%)			0.879
II	2 (2.9)	1 (3.3)	
IIIa	13 (18.8)	5 (16.7)	
IIIb	31 (44.9)	16 (53.3)	
IV	23 (33.3)	8 (26.7)	
Liver resection type, *n* (%)			0.817
Right hepatectomy + S1	14 (20.3)	5 (16.7)	
Right trisectionectomy + S1	3 (4.3)	1 (3.3)	
Left hepatectomy + S1	50 (72.5)	22 (73.3)	
Left trisectionectomy + S1	2 (2.9)	2 (6.7)	
Estimated blood loss, mL	400 (300–500)	400 (300–600)	0.455
Intraoperative transfusion, *n* (%)	22 (31.9)	5 (16.7)	0.118
Main portal vein resection and reconstruction, *n* (%)	3 (4.4)	2 (6.7)	0.637
Portal occlusion >15 min, *n* (%)	25 (36.2)	10 (33.3)	0.782
Operation time, min	300 (280–320)	320 (280–360)	0.140
Fibrosis Laennec staging, *n* (%)			0.038*
F0	8 (11.6)	9 (30.0)	
F1	17 (24.6)	10 (33.3)	
F2	27 (39.1)	10 (33.3)	
F3	15 (21.7)	1 (3.3)	
F4/cirrhosis	2 (2.9)	0 (0)	
Significant fibrosis (F2 + F3 + F4)	44 (63.8)	11 (36.7)	0.016*
Positive surgical margin, *n* (%)	5 (7.3)	3 (10.0)	0.695
Microvascular invasion, *n* (%)	8 (11.6)	5 (16.7)	0.492
Perineural invasion, *n* (%)	9 (13.0)	7 (23.3)	0.201
Lymph node metastasis, *n* (%)	22 (31.8)	11 (36.7)	0.643
Histological differentiation, *n* (%)			0.206
Well	12 (17.4)	10 (14.5)	
Moderate	32 (46.4)	12 (17.4)	
Poor	25 (36.2)	8 (11.6)	
*Postoperative data*			
Overall complication, *n* (%)	31 (44.9)	6 (20.0)	0.018*
Postoperative bleeding, *n* (%)	3 (4.4)	1 (3.3)	1.000
Bile leakage, *n* (%)	3 (4.4)	1 (3.3)	1.000
Infection, *n* (%)	6 (8.7)	2 (6.7)	1.000
Massive ascites, *n* (%)	18 (26.1)	2 (6.7)	0.030*
Pleural effusion, *n* (%)	8 (11.6)	2 (6.7)	0.719
Liver failure, *n* (%)	11 (15.9)	1 (3.3)	0.077
Hospital days, days	23 (20–25)	20 (18–22)	0.012*
90‐days mortality, *n* (%)	7 (10.1)	1 (3.3)	0.429

Abbreviations: ALT, alanine transaminase; Alb, albumin; CA19‐9, carbohydrate antigen 19–9; CEA, carcinoembryonic antigen; EBD, endoscopic biliary drainage; HB, hemoglobin; HBcAb, hepatitis B core antibody; INR, international normalized ratio; PT, prothrombin time; PTCD, percutaneous transhepatic cholangial drainage; TB, total bilirubin.

* *p* Difference was statistically significant.

Pathological analysis of liver fibrosis was performed in 99 patients enrolled in the study. The pathological staging of liver fibrosis was summarized according to the Laennec staging system; the incidence of cirrhosis (F4) in all patients was 2.0% (2/99). The rate of significant fibrosis (F2 + F3 + F4) in the HBcAb+ group (44/69, 63.8%) was significantly higher than that in the HBcAb‐ group (11/30, 36.7%) (*p* = 0.016). Advanced fibrosis (F3 + F4) was detected in 24.6% of the patients with HBcAb‐positive, which was markedly higher than those with HBcAb‐negative (3.3%) (*p* = 0.011). Overall, HBcAb‐positive patients were more likely to develop more severe fibrosis or cirrhosis.

As shown in Table 1, the incidences of postoperative complications (*p* = 0.014) and massive ascites (*p* = 0.035) in the HBcAb+ group were significantly higher than those in the HBcAb‐ group (*p* < 0.05). Although not statistically significant, the incidence of liver failure (*p* = 0.077) in the HBcAb+ group (15.9%) was higher than that in the HBcAb‐ group (3.3%). The length of stay in hospital was significantly longer in the HBcAb+ group compared to in the HBcAb‐ group (*p* = 0.012). There was no statistically significant difference between the two groups in the surgical procedures and other postoperative complications (*p* > 0.05).

Most patients who died within 90‐day after surgery were HBcAb‐positive, the leading causes of death were liver failure (six cases) and hemorrhagic shock (three cases), and all the patients who died within 90‐day after surgery had significant liver fibrosis (F2/F3/F4).

Univariate and multivariate analyses of risk factors for complications were performed by logistics regression, and the results were summarized in Table 2. Multivariate analysis indicated that HBcAb positivity (adjusted odds ratio [OR], 4.534; 95% confidence interval [95% CI], 1.190–17.28; *p* = 0.027), preoperative cholangitis (OR, 8.179; 95% CI, 2.164–30.92; *p* = 0.002), portal occlusion >15 min (OR, 4.671; 95% CI 1.592–17.10; *p* = 0.005), significant fibrosis (OR, 3.609; 95% CI 1.142–11.41; *p* = 0.029) were independent risk factors for complications of hCCA patients after extended hemihepatectomy.

**TABLE 2:** Univariate and multivariate analysis of postoperative complications with hilar cholangiocarcinoma.

Variable (yes/no)	OR	95%CI	*p* value
*Univariate analysis*			
Age > 60 years	1.107	0.476–2.573	0.814
Sex, male	1.003	0.440–2.313	0.983
HBcAb positivity	3.263	1.185–8.982	0.018*
Jaundice	1.082	0.422–2.776	0.870
Caroli disease	1.134	0.653–3.453	0.179
Primary sclerosing cholangitis	1.227	0.717–3.167	0.224
Choledochal cysts	1.315	0.734–2.926	0.133
Hepatolithiasis	1.442	0.489–3.626	0.174
Alcohol drinking	1.257	0.738–3.234	0.125
Preoperative cholangitis	6.395	2.392–17.09	<0.001*
TB >34 μmol/L	0.907	0.348–2.366	0.842
Preoperative biliary drainage	1.193	0.483–2.947	0.702
Hb >120 g/L	0.893	0.363–2.194	0.805
PT >1.20 INR	0.259	0.030–2.244	0.252
ALT >40 U/L	1.273	0.502–3.225	0.611
Alb >35 g/L	1.057	0.395–2.827	0.912
CA 19–9 > 37 U/mL	0.994	0.329–3.001	0.991
Bismuth‐Corlett type VI	0.888	0.367–2.149	0.793
Hepatectomy, right‐sided	1.763	0.685–4.538	0.237
Estimated blood loss >500 mL	1.270	0.497–3.246	0.617
Intraoperative transfusion	0.624	0.241–1.616	0.329
Revascularization	1.124	0.179–7.058	0.901
Portal occlusion >15 min	6.845	2.740–17.10	<0.001*
Operation time >300 min	1.462	0.645–3.314	0.363
Positive surgical margin	1.758	0.412–7.495	0.441
Significant fibrosis	3.279	1.358–7.914	0.007*
*Multivariate analysis*			
HBcAb positivity	4.533	1.299–19.68	0.027*
Cholangitis	8.179	2.329–34.80	0.002*
Portal occlusion >15 min	4.670	1.623–14.26	0.005*
Significant fibrosis	3.610	1.200–12.48	0.029*

Abbreviations: Alb, albumin; ALT, alanine transaminase; CA19‐9, carbohydrate antigen 19–9; CEA, carcinoembryonic antigen; CI, confidence interval; HB, hemoglobin; HBcAb, hepatitis B core antibody; INR, international normalized ratio; OR, odds ratio; PT, prothrombin time; TB, total bilirubin.

* *p* Difference was statistically significant.

### 3.3 Long‐term survival after liver resection

**FIGURE 2:**
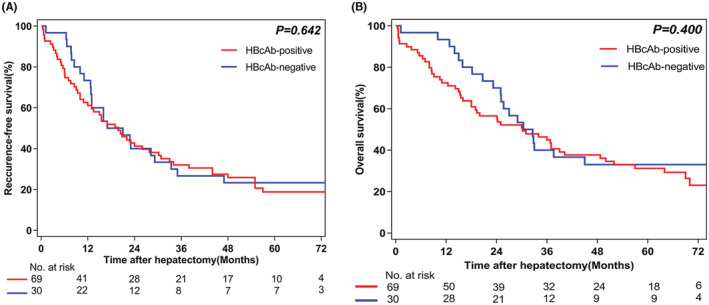
Recurrence‐free survival (RFS) and overall survival (OS) rates of hepatitis B core antibody (HBcAb)‐positive versus HBcAb‐negative for hilar cholangiocarcinoma (hCCA) patients. (A) RFS and (B) OS curves of HBcAb‐positive (*n* = 69) and HBcAb‐negative (*n* = 30) groups (*p* = 0.642 and *p* = 0.400, respectively).

We followed up to December 31, 2021, and the median follow‐up period was 68 months (range 0.6–96 months). The 1‐, 3‐, and 5‐year recurrence‐free survival (RFS) rates for all 99 patients were 64.9%, 30.3%, and 20.4%, respectively. The 1‐, 3‐, and 5‐year OS rates for all 99 patients were 77.7%, 43.4%, and 31.8%, respectively. Furthermore, the RFS and OS rates were 18.8% and 31.2% in the HBcAb+ group, 23.3% and 33.0% in the HBcAb‐ group at 5 years, respectively. There were no significant differences in RFS and OS between HBcAb‐positive and HBcAb‐negative patients (*p* = 0.642 and *p* = 0.400, respectively) (Figure 2A,B).

In order to identify prognostic risk factors in patients with hCCA who underwent liver resection, Cox univariate and multivariate analyses were performed. Multivariate analysis indicated that positive surgical margin [adjusted hazard ratio (HR), 3.341; 95% CI, 1.528–7.306; *p* = 0.003], microvascular invasion (HR, 2.720; 95% CI, 1.453–5.095; *p* = 0.002), lymph node metastasis (HR, 2.573; 95% CI, 1.573–4.209; *p* < 0.001) were independent risk factors for recurrence‐free survival. Positive surgical margin (HR 2.365, 95% CI 1.085–5.151; *p* = 0.030), microvascular invasion (HR, 2.705; 95% CI, 1.384–5.287; *p* = 0.004), lymph node metastasis (HR, 2.573; 95% CI, 1.312–3.602; *p* = 0.003), poor differentiation (HR 1.876, 95% CI 1.160–3.035; *p* = 0.010) were independent risk factors for OS (Table S1). Kaplan–Meier survival curves of surgical margin, microvascular invasion, lymph node metastasis, and pathological differentiation are shown in Figure S1.

## 4 DISCUSSION

To the best of our knowledge, this is the first study to demonstrate the incidence of HBcAb positivity in hCCA patients and the effect of HBcAb positivity on surgical complications for hCCA. We found that HBcAb positivity was common in hCCA patients (73.7%), and HBcAb positivity significantly increased the incidence of postoperative complications after extended hemihepatectomy for hCCA.

China has a high prevalence of HBV infection, where 120 million cases are infected with chronic HBV, and about 300,000 patients die from HBV infection‐related diseases (e.g., cirrhosis, liver failure, carcinoma, etc.) annually.^25^ The epidemiological serosurvey of HBV infection conducted in 2006 in China showed that the positive rates of HBsAg, anti‐HBs, and anti‐HBc in Chinese cases aged 1–59 years old were 7.2%, 50.1%, and 34.1%, respectively.^13^ The present research revealed that the prevalence rates of HBsAg, HBsAb, and HBcAb in hCCA patients were 10.6%, 48.4%, and 73.7%, respectively. The prevalence of HBcAb positivity in hCCA patients was remarkably higher than that in healthy individuals, suggesting a close relationship between hCCA and HBcAb positivity.

Numerous studies demonstrated that HBV infection was closely associated with hepatocellular carcinoma (HCC) and CCA.^26^, ^27^, ^28^, ^29^, ^30^ Although the exact effect of HBV on tumorigenesis is unknown, integration of HBV DNA into the host genome induces genomic instability and direct insertion mutations of multiple cancer‐related genes, which have been proven to be an essential risk factor for carcinogenesis.^28^, ^30^ As we all know, both hepatocytes and cholangiocytes have the same progenitor cells. Therefore, HBV is likely to induce HCC and CCA through similar mechanisms.^31^ In addition, inflammatory changes caused by the continuous presence of the virus involve multiple signaling pathways leading to malignant transformation.^29^ HBcAb positivity reflects prior HBV infection, which has recently attracted attention as a risk factor for liver carcinogenesis.^32^, ^33^, ^34^ A previous meta‐analysis suggested that HBcAb positivity increased the risk of cholangiocarcinoma.^32^ However, no study has focused on surgical safety for hCCA in HBcAb‐positive patients.

The current study revealed that the stage of liver fibrosis in HBcAb‐positive patients was significantly higher than that in HBcAb‐negative patients, and advanced fibrosis or significant fibrosis was more prevalent in HBcAb‐positive than in HBcAb‐negative patients. Previous studies have demonstrated an increased prevalence of advanced fibrosis/cirrhosis in prior hepatitis B patients, consistent with the present study's findings.^35^, ^36^ Liver fibrosis is characterized by excessive accumulation of extracellular matrix, which distorts the physiological architecture of the liver.^37^ Severe fibrosis or cirrhosis would impair the synthetic and metabolic functions of the liver, and it might involve a higher potential surgical risk.^38^, ^39^ HBcAb positivity can not only cause liver fibrosis and then decrease reserve function but also cause inflammatory liver injury, which can affect liver function. Therefore, HBcAb positivity and fibrosis have overlapping effects on liver reserve function. Still, HBcAb positivity could act as an independent factor, mainly because prior HBV infection can also impair liver reserve function through hepatitis, hepatocyte injury, virus reactivation, and other ways, thus affecting surgical safety.^28^, ^35^, ^40^

Hemihepatectomy/trisectionectomy has widely been advocated as the preferred surgical treatment for hCCA, which has the advantage of obtaining a wide and negative surgical margin and thus improving the surgical outcomes.^41^, ^42^ Considering that hilar cholangiocarcinoma is likely to invade the parenchyma and the biliary branches of the caudate lobe, total caudate lobectomy was carried out for the radical treatment of hCCA.^42^, ^43^ Meanwhile, all patients underwent lymph node dissection and biliary reconstruction in our study. According to the NCCN guideline, liver resection is a potentially curative and safe option when the future remnant liver volume to the total liver volume ranges from 20% to 30% for patients with normal liver.^39^, ^44^ Hepatitis infection and severe liver fibrosis may affect liver functional reserve and postoperative remnant liver regeneration.^45^, ^46^ When the residual liver volume is insufficient to maintain the physiological function of the liver, it can lead to coagulation disorders, massive ascites, liver failure, severe infection, acute respiratory and circulatory failure, and even death.^47^, ^48^, ^49^ Thus, preserving more liver parenchyma for patients with liver injury due to hepatitis infection or fibrosis is more critical to those receiving major liver resection because postoperative complication is a primary concern.^50^ In the present study, the patients underwent surgery when the estimated residual liver volume was greater than 30%, and routine preoperative examinations were consistent with surgical indications. In the present study, the incidence of postoperative complications and massive ascites in HBcAb‐positive patients were significantly higher than those in HBcAb‐negative patients; similarly, the incidence of liver failure in the HBcAb‐positive group (14.5%) was higher than that in the HBcAb‐negative group (3.3%). In addition, HBcAb‐positive patients had higher mortality within 30‐day after surgery than HBcAb‐negative patients (7.2% vs. 0%). Multivariate analysis indicated that the HBcAb positivity was a significant independent risk factor of postoperative complications (OR 4.534, 95% CI 1.19–17.28, *p* = 0.027). Moreover, liver fibrosis was another independent risk factor for postoperative complications, which might reflect prior HBV infection directly or indirectly caused liver damage and thereby increased the incidence of postoperative complications. Therefore, HBcAb positivity markedly increased the incidence of postoperative complications after liver resection for hCCA.

Previous studies on the relationship between HBV infection and the postoperative prognosis of iCCA are still controversial. Some reports suggested that current or previous HBV infection could improve the prognosis of iCCA,^51^, ^52^ while other studies indicated that HBV infection was associated with a worse prognosis.^53^ However, no research has investigated whether HBV infection or HBcAb positivity will affect the prognosis of hCCA patients. In our study, another important finding was that there was no significant difference in OS or RFS between HBcAb‐positive and HBcAb‐negative patients, so the long‐term prognosis of hCCA patients was not correlated with HBcAb status. Multivariate analyses revealed that the prognosis of hCCA is determined primarily by the more aggressive tumor and insignificantly by the status of HBcAb.

To ensure the R0 resection of hCCA while reducing the incidence of postoperative complications, we previously found that^54^ hCCA had limited axial invasion. Thus, adjusting surgical strategies such as decreasing the extent of liver resection may provide a more safe and effective treatment for HBcAb‐positive patients. In other words, reducing the volume of liver resection can be an appropriate solution for high‐risk hCCA patients associated with underlying diseases such as prior HBV infection.

This study had some limitations. First, this was a single‐center retrospective study with a small sample, meaning there is a high chance of it having selection bias. Second, heterogeneity of the included studies was induced by some factors, such as different surgical procedures. However, due to the small sample, we did not conduct subgroup analyses of surgical procedures. Third, we have not analyzed the impact of postoperative therapy on the long‐term outcomes due to unavailable data. More prospective studies with a large sample size and RCT studies should be further carried out to explore the impact of HBcAb status on surgical outcomes of hCCA patients.

## 5 CONCLUSIONS

In summary, HBcAb positivity is a common phenomenon in hCCA patients from China. HBcAb positivity markedly increased the risk of postoperative complications and mortality after extended hemihepatectomy for hCCA. Consequently, hepatobiliary surgeons should pay more attention to HBcAb status in hCCA patients with negative HBsAg, which could help them to select optimal surgical modalities.

## AUTHOR CONTRIBUTIONS

**Wen‐Qiang Wang:** Conceptualization (equal); data curation (equal); formal analysis (lead); methodology (lead); validation (equal); writing – original draft (lead). **Guang‐Yuan Xu:** Conceptualization (equal); data curation (equal); software (equal); validation (equal). **Jian Li:** Investigation (equal); software (equal). **Bin‐Yong Liang:** Resources (equal); validation (equal). **jiang Li:** Methodology (equal); visualization (equal). **Mei‐Long Lin:** Investigation (equal). **Xiao‐Ping Chen:** Supervision (equal). **Er‐Lei Zhang:** Validation (equal); writing – original draft (equal); writing – review and editing (lead). **Zhi‐Yong huang:** Project administration (lead); supervision (lead); writing – original draft (equal); writing – review and editing (lead).

## FUNDING INFORMATION

This study was supported by Hubei provincial special grants for scientific and technical innovation (No. 2021BCA115) and National Natural Science Foundation of China (No. 81902839).

## CONFLICT OF INTEREST STATEMENT

The authors declare that they have no conflicts of interest.

## ETHICS STATEMENT

This retrospective study protocol was approved by the Ethics Committee of Tongji Medical College, Huazhong University of Science and Technology (2020‐S293). Written informed consent from each patient was waived for this study because not impacting patient management.

## Supporting information


Supplementary Table S1.

## Data Availability

The data that support the findings of this study are available from the corresponding author Zhi Yong Huang, upon reasonable request; in order to preserve patient privacy.

## References

[1] Razumilava N , Gores GJ . Cholangiocarcinoma. Lancet. 2014;383:2168‐2179. 24581682 10.1016/S0140-6736(13)61903-0 PMC4069226

[2] Blechacz B , Gores GJ . Cholangiocarcinoma: advances in pathogenesis, diagnosis, and treatment. Hepatology. 2008;48:308‐321. 18536057 10.1002/hep.22310 PMC2547491

[3] Khan SA , Tavolari S , Brandi G . Cholangiocarcinoma: epidemiology and risk factors. Liver Int. 2019;39(Suppl 1):19‐31. 30851228 10.1111/liv.14095

[4] Geers J , Jaekers J , Topal H , et al. Predictors of survival after surgery with curative intent for perihilar cholangiocarcinoma. World J Surg Oncol. 2020;18:286. 33143698 10.1186/s12957-020-02060-x PMC7641817

[5] Unno M , Katayose Y , Rikiyama T , et al. Major hepatectomy for perihilar cholangiocarcinoma. J Hepatobiliary Pancreat Sci. 2010;17:463‐469. 19941010 10.1007/s00534-009-0206-3

[6] Nagino M , Ebata T , Yokoyama Y , et al. Evolution of surgical treatment for perihilar cholangiocarcinoma: a single‐center 34‐year review of 574 consecutive resections. Ann Surg. 2013;258:129‐140. 23059502 10.1097/SLA.0b013e3182708b57

[7] Seyama Y , Kubota K , Sano K , et al. Long‐term outcome of extended hemihepatectomy for hilar bile duct cancer with no mortality and high survival rate. Ann Surg. 2003;238:73‐83. 12832968 10.1097/01.SLA.0000074960.55004.72 PMC1422671

[8] de Jong MC , Marques H , Clary BM , et al. The impact of portal vein resection on outcomes for hilar cholangiocarcinoma: a multi‐institutional analysis of 305 cases. Cancer. 2012;118:4737‐4747. 22415526 10.1002/cncr.27492

[9] Nuzzo G . Improvement in perioperative and long‐term outcome after surgical treatment of hilar cholangiocarcinoma. Arch Surg. 2012;147:26‐34. 22250108 10.1001/archsurg.2011.771

[10] Terrault NA , Lok ASF , McMahon BJ , et al. Update on prevention, diagnosis, and treatment of chronic hepatitis B: AASLD 2018 hepatitis B guidance. Hepatology. 2018;67:1560‐1599. 29405329 10.1002/hep.29800 PMC5975958

[11] Yeo W , Johnson PJ . Diagnosis, prevention and management of hepatitis B virus reactivation during anticancer therapy. Hepatology. 2006;43:209‐220. 16440366 10.1002/hep.21051

[12] EASL . Clinical practice guidelines on the management of hepatitis B virus infection. J Hepatol. 2017;2017(67):370‐398. 10.1016/j.jhep.2017.03.021 28427875

[13] Liang X , Bi S , Yang W , et al. Epidemiological serosurvey of hepatitis B in China—declining HBV prevalence due to hepatitis B vaccination. Vaccine. 2009;27:6550‐6557. 19729084 10.1016/j.vaccine.2009.08.048

[14] Luo Z , Li L , Ruan B . Impact of the implementation of a vaccination strategy on hepatitis B virus infections in China over a 20‐year period. Int J Infect Dis. 2012;16:e82‐e88. 22178658 10.1016/j.ijid.2011.10.009

[15] Clements O , Eliahoo J , Kim JU , Taylor‐Robinson SD , Khan SA . Risk factors for intrahepatic and extrahepatic cholangiocarcinoma: a systematic review and meta‐analysis. J Hepatol. 2020;72:95‐103. 31536748 10.1016/j.jhep.2019.09.007

[16] Tousif K , Nicholas LS , Zoe ZXT , et al. Predictors of post‐operative complications after surgical resection of hepatocellular carcinoma and their prognostic effects on outcome and survival: a propensity‐score matched and structural equation modelling study. Eur J Surg Oncol. 2020;46:1756‐1765. 32345496 10.1016/j.ejso.2020.03.219

[17] Bismuth H , Nakache R , Diamond T . Management strategies in resection for hilar cholangiocarcinoma. Ann Surg. 1992;215:31‐38. 1309988 10.1097/00000658-199201000-00005 PMC1242367

[18] Rastogi A , Maiwall R , Bihari C , et al. Cirrhosis histology and Laennec staging system correlate with high portal pressure. Histopathology. 2013;62:731‐741. 23470026 10.1111/his.12070

[19] Chan KM , Lee CF , Wu TJ , et al. Adverse outcomes in patients with postoperative ascites after liver resection for hepatocellular carcinoma. World J Surg. 2012;36:392‐400. 22131090 10.1007/s00268-011-1367-1

[20] Moore KP , Wong F , Gines P , et al. The management of ascites in cirrhosis: report on the consensus conference of the international ascites club. Hepatology. 2003;38:258‐266. 12830009 10.1053/jhep.2003.50315

[21] Ishizawa T , Hasegawa K , Kokudo N , et al. Risk factors and management of ascites after liver resection to treat hepatocellular carcinoma. Arch Surg. 2009;144:46‐51. 19153324 10.1001/archsurg.2008.511

[22] Nguyen HB , Rivers EP , Abrahamian FM , et al. Severe sepsis and septic shock: review of the literature and emergency department management guidelines. Ann Emerg Med. 2006;48:28‐54. 16781920 10.1016/j.annemergmed.2006.02.015

[23] Koch M , Garden OJ , Padbury R , et al. Bile leakage after hepatobiliary and pancreatic surgery: a definition and grading of severity by the international study group of liver surgery. Surgery. 2011;149:680‐688. 21316725 10.1016/j.surg.2010.12.002

[24] Rahbari NN , Garden OJ , Padbury R , et al. Posthepatectomy liver failure: a definition and grading by the international study group of liver surgery (ISGLS). Surgery. 2011;149:713‐724. 21236455 10.1016/j.surg.2010.10.001

[25] Ott JJ , Stevens GA , Groeger J , Wiersma ST . Global epidemiology of hepatitis B virus infection: new estimates of age‐specific HBsAg seroprevalence and endemicity. Vaccine. 2012;30:2212‐2219. 22273662 10.1016/j.vaccine.2011.12.116

[26] Li M , Li J , Li P , et al. Hepatitis B virus infection increases the risk of cholangiocarcinoma: a meta‐analysis and systematic review. J Gastroenterol Hepatol. 2012;27:1561‐1568. 22694354 10.1111/j.1440-1746.2012.07207.x

[27] Levrero M , Zucman‐Rossi J . Mechanisms of HBV‐induced hepatocellular carcinoma. J Hepatol. 2016;64:S84‐S101. 27084040 10.1016/j.jhep.2016.02.021

[28] Trépo C , Chan HL , Lok A . Hepatitis B virus infection. Lancet. 2014;384:2053‐2063. 24954675 10.1016/S0140-6736(14)60220-8

[29] Fragkou N , Sideras L , Panas P , Emmanouilides C , Sinakos E . Update on the association of hepatitis B with intrahepatic cholangiocarcinoma: is there new evidence? World J Gastroenterol. 2021;27:4252‐4275. 34366604 10.3748/wjg.v27.i27.4252 PMC8316913

[30] Bonilla Guerrero R , Roberts LR . The role of hepatitis B virus integrations in the pathogenesis of human hepatocellular carcinoma. J Hepatol. 2005;42:760‐777. 15826727 10.1016/j.jhep.2005.02.005

[31] Lee CH , Chang CJ , Lin YJ , Yeh CN , Chen MF , Hsieh SY . Viral hepatitis‐associated intrahepatic cholangiocarcinoma shares common disease processes with hepatocellular carcinoma. Br J Cancer. 2009;100:1765‐1770. 19436294 10.1038/sj.bjc.6605063 PMC2695699

[32] Zhang H , Zhu B , Zhang H , Liang J , Zeng W . HBV infection status and the risk of cholangiocarcinoma in Asia: a meta‐analysis. Biomed Res Int. 2016;2016:3417976. 27999794 10.1155/2016/3417976 PMC5141322

[33] Okamura Y , Sugiura T , Ito T , Yamamoto Y , Ashida R , Uesaka K . The impact of the hepatitis B core antibody status on recurrence in patients with non‐B non‐C hepatocellular carcinoma after curative surgery. Dig Surg. 2018;35:243‐251. 28810252 10.1159/000479340

[34] Omichi K , Shindoh J , Yamamoto S , et al. Postoperative outcomes for patients with non‐B non‐C hepatocellular carcinoma: a subgroup analysis of patients with a history of hepatitis B infection. Ann Surg Oncol. 2015;22(Suppl 3):S1034‐S1040. 26350363 10.1245/s10434-015-4845-0

[35] Torbenson M , Thomas DL . Occult hepatitis B. Lancet Infect Dis. 2002;2:479‐486. 12150847 10.1016/s1473-3099(02)00345-6

[36] Covolo L , Pollicino T , Raimondo G , Donato F . Occult hepatitis B virus and the risk for chronic liver disease: a meta‐analysis. Dig Liver Dis. 2013;45:238‐244. 23146778 10.1016/j.dld.2012.09.021

[37] Hernandez‐Gea V , Friedman SL . Pathogenesis of liver fibrosis. Annu Rev Pathol. 2011;6:425‐456. 21073339 10.1146/annurev-pathol-011110-130246

[38] Cai S , Ou Z , Liu D , et al. Risk factors associated with liver steatosis and fibrosis in chronic hepatitis B patient with component of metabolic syndrome. United European Gastroenterol J. 2018;6:558‐566. 10.1177/2050640617751252 PMC5987283 29881611

[39] Guglielmi A , Ruzzenente A , Conci S , Valdegamberi A , Iacono C . How much remnant is enough in liver resection? Dig Surg. 2012;29:6‐17. 22441614 10.1159/000335713

[40] Suhail M , Abdel‐Hafiz H , Ali A , et al. Potential mechanisms of hepatitis B virus induced liver injury. World J Gastroenterol. 2014;20:12462‐12472. 25253946 10.3748/wjg.v20.i35.12462 PMC4168079

[41] Lauterio A , De Carlis R , Centonze L , et al. Current surgical management of peri‐hilar and intra‐hepatic cholangiocarcinoma. Cancers (Basel). 2021;13(15):3657. 34359560 10.3390/cancers13153657 PMC8345178

[42] Mansour JC , Aloia TA , Crane CH , Heimbach JK , Nagino M , Vauthey JN . Hilar cholangiocarcinoma: expert consensus statement. HPB (Oxford). 2015;17:691‐699. 26172136 10.1111/hpb.12450 PMC4527854

[43] Xiang S , Lau WY , Chen XP . Hilar cholangiocarcinoma: controversies on the extent of surgical resection aiming at cure. Int J Colorectal Dis. 2015;30:159‐171. 25376337 10.1007/s00384-014-2063-z PMC4304009

[44] Benson AB , D'Angelica MI , Abbott DE , et al. Hepatobiliary cancers, version 2.2021, NCCN Clinical Practice Guidelines in Oncology. J Natl Compr Canc Netw. 2021;19:541‐565. 34030131 10.6004/jnccn.2021.0022

[45] Fausto N , Campbell JS , Riehle KJ . Liver regeneration. Hepatology. 2006;43:S45‐S53. 16447274 10.1002/hep.20969

[46] Agrawal S , Belghiti J . Oncologic resection for malignant tumors of the liver. Ann Surg. 2011;253:656‐665. 21475004 10.1097/SLA.0b013e3181fc08ca

[47] Tsuchikawa T , Hirano S , Okamura K , et al. Advances in the surgical treatment of hilar cholangiocarcinoma. Expert Rev Gastroenterol Hepatol. 2015;9:369‐374. 25256146 10.1586/17474124.2015.960393

[48] van Gulik TM , Ruys AT , Busch OR , Rauws EA , Gouma DJ . Extent of liver resection for hilar cholangiocarcinoma (Klatskin tumor): how much is enough? Dig Surg. 2011;28:141‐147. 21540600 10.1159/000323825

[49] Bernal W , Lee WM , Wendon J , Larsen FS , Williams R . Acute liver failure: a curable disease by 2024? J Hepatol. 2015;62:S112‐S120. 25920080 10.1016/j.jhep.2014.12.016

[50] Zhou SJ , Zhang EL , Liang BY , et al. Morphologic severity of cirrhosis determines the extent of liver resection in patients with hepatocellular carcinoma and child‐Pugh grade a cirrhosis. J Surg Res. 2016;200:444‐451. 26470819 10.1016/j.jss.2015.08.027

[51] Zhou HB , Wang H , Li YQ , et al. Hepatitis B virus infection: a favorable prognostic factor for intrahepatic cholangiocarcinoma after resection. World J Gastroenterol. 2011;17:1292‐1303. 21455328 10.3748/wjg.v17.i10.1292 PMC3068264

[52] Liu RQ , Shen SJ , Hu XF , Liu J , Chen LJ , Li XY . Prognosis of the intrahepatic cholangiocarcinoma after resection: hepatitis B virus infection and adjuvant chemotherapy are favorable prognosis factors. Cancer Cell Int. 2013;13:99. 24139471 10.1186/1475-2867-13-99 PMC3852727

[53] Lei Z , Xia Y , Si A , et al. Antiviral therapy improves survival in patients with HBV infection and intrahepatic cholangiocarcinoma undergoing liver resection. J Hepatol. 2018;68:655‐662. 29155069 10.1016/j.jhep.2017.11.015

[54] Chen XP , Lau WY , Huang ZY , et al. Extent of liver resection for hilar cholangiocarcinoma. Br J Surg. 2009;96:1167‐1175. 19705374 10.1002/bjs.6618

