# Supplementary material for: HBcAb positivity increases the risk of postoperative complications after extended hemihepatectomy for hilar cholangiocarcinoma
Source: Cancer Med. 2023 Feb 27;12(8):9627–36. doi: 10.1002/cam4.5740 (PMC10166974; doi:10.1002/cam4.5740)
Supplement: Supplementary file 1 — Supplementary Table S1. [file CAM4-12-9627-s001.docx]

| Variable (Yes/No) | RFS |  |  | OS |  |  |
| --- | --- | --- | --- | --- | --- | --- |
|  | HR | 95%CI | P value | HR | 95%CI | P value |
| **Univariate analysis** | | | | | | |
| Age >60 years | 1.580 | 1.001-2.496 | 0.050 | 1.328 | 0.823-2.144 | 0.245 |
| Sex, male | 0.636 | 0.403-1.002 | 0.051 | 0.735 | 0.459-1.179 | 0.202 |
| HBcAb | 1.122 | 0.688-1.832 | 0.644 | 1.248 | 0.743-2.095 | 0.400 |
| Jaundice | 0.676 | 0.407-1.122 | 0.130 | 0.622 | 0.368-1.050 | 0.075 |
| Caroli disease | 1.733 | 0.653-3.453 | 0.138 | 1.542 | 0.676-3.022 | 0.331 |
| Primary sclerosing cholangitis | 1.566 | 0.717-3.167 | 0.217 | 1.345 | 0.589-2.977 | 0.521 |
| Choledochal cysts | 1.586 | 0.734-2.926 | 0.433 | 1.672 | 0.644-2.568 | 0.314 |
| Hepatolithiasis | 2.255 | 0.489-3.626 | 0.445 | 2.132 | 0.283-3.112 | 0.532 |
| Alcohol drinking | 1.321 | 0.738-3.234 | 0.311 | 1.457 | 0.533-3.101 | 0.277 |
| Preoperative cholangitis | 1.491 | 0.911-2.441 | 0.112 | 1.696 | 1.015-2.832 | 0.044 |
| Preoperative biliary drainage | 0.679 | 0.416-1.108 | 0.121 | 0.588 | 0.356-0.970 | 0.037 |
| TB >34 μmol/L | 0.743 | 0.440-1.252 | 0.264 | 0.692 | 0.403-1.188 | 0.182 |
| Hb <120 g/L | 1.490 | 0.877-2.531 | 0.140 | 1.286 | 0.753-2.197 | 0.357 |
| PT >1.20 INR | 1.314 | 0.570-3.028 | 0.522 | 1.151 | 0.463-2.862 | 0.762 |
| ALT >40 U/L | 0.990 | 0.603-1.626 | 0.968 | 1.022 | 0.604-1.729 | 0.934 |
| Alb <35 g/L | 0.912 | 0.542-1.534 | 0.729 | 0.979 | 0.567-1.690 | 0.940 |
| CEA >5 ng/ml | 1.187 | 0.751-1.875 | 0.462 | 1.225 | 0.764-1.964 | 0.399 |
| CA19-9 >37 U/ml | 1.104 | 0.596-2.046 | 0.753 | 0.989 | 0.523-1.895 | 0.996 |
| Bismuth-Corlett type VI | 1.017 | 0.630-1.641 | 0.946 | 1.046 | 0.636-1.721 | 0.860 |
| Hepatectomy, right-sided | 1.349 | 0.800-2.276 | 0.261 | 1.370 | 0.808-2.322 | 0.243 |
| Estimated blood loss >500ml | 1.194 | 0.715-1.992 | 0.498 | 1.138 | 0.665-1.946 | 0.637 |
| Intraoperative transfusion | 1.535 | 0.938-2.510 | 0.088 | 1.148 | 0.687-1.920 | 0.598 |
| Revascularization | 1.766 | 0.642-4.858 | 0.271 | 1.717 | 0.623-4.732 | 0.296 |
| Portal occlusion >15min | 1.376 | 0.860-2.204 | 0.183 | 1.469 | 0.909-2.375 | 0.117 |
| Operation time >300min | 0.881 | 0.559-1.387 | 0.584 | 0.939 | 0.587-1.503 | 0.795 |
| High-grade fibrosis | 1.433 | 0.907-2.263 | 0.123 | 1.657 | 1.025-2.681 | 0.039 |
| Positive surgical margin | 4.116 | 1.882-9.000 | <0.001 | 2.886 | 1.343-6.202 | 0.004 |
| Microvascular invasion | 2.460 | 1.335-4.534 | 0.003 | 2.245 | 1.166-4.322 | 0.013 |
| Perineural invasion | 1.517 | 0.861-2.674 | 0.149 | 1.686 | 0.945-3.010 | 0.077 |
| Lymph node metastasis | 2.420 | 1.497-3.912 | <0.001 | 2.227 | 1.363-3.639 | <0.001 |
| Tumor differentiation, poor | 1.725 | 1.080-2.756 | 0.020 | 1.896 | 1.180-3.046 | 0.007 |
| **Multivariate analysis** | | | | | | |
| Positive surgical margin | 3.341 | 1.528-7.306 | 0.003 | 2.365 | 1.085-5.151 | 0.030 |
| Microvascular invasion | 2.720 | 1.453-5.095 | 0.002 | 2.705 | 1.384-5.287 | 0.004 |
| Lymph node metastasis | 2.573 | 1.573-4.209 | <0.001 | 2.174 | 1.312-3.602 | 0.003 |
| Tumor differentiation, poor | - | - | - | 1.876 | 1.160-3.035 | 0.010 |

**Supplementary Table S1.** Univariate and multivariate analysis of prognostic factors in patients with hilar cholangiocarcinoma

*RFS,* recurrence-free survival*; OS,* overall survival*; HR*, hazards ratio; *CI*, confidence interval; *HBcAb*, hepatitis B core antibody; *TB*, total bilirubin; *HB*, hemoglobin; *PT*, prothrombin time; *INR,* international normalized ratio; *ALT*, alanine transaminase; *Alb*, albumin; CEA, carcinoembryonic antigen; *CA19-9*, carbohydrate antigen 19-9.


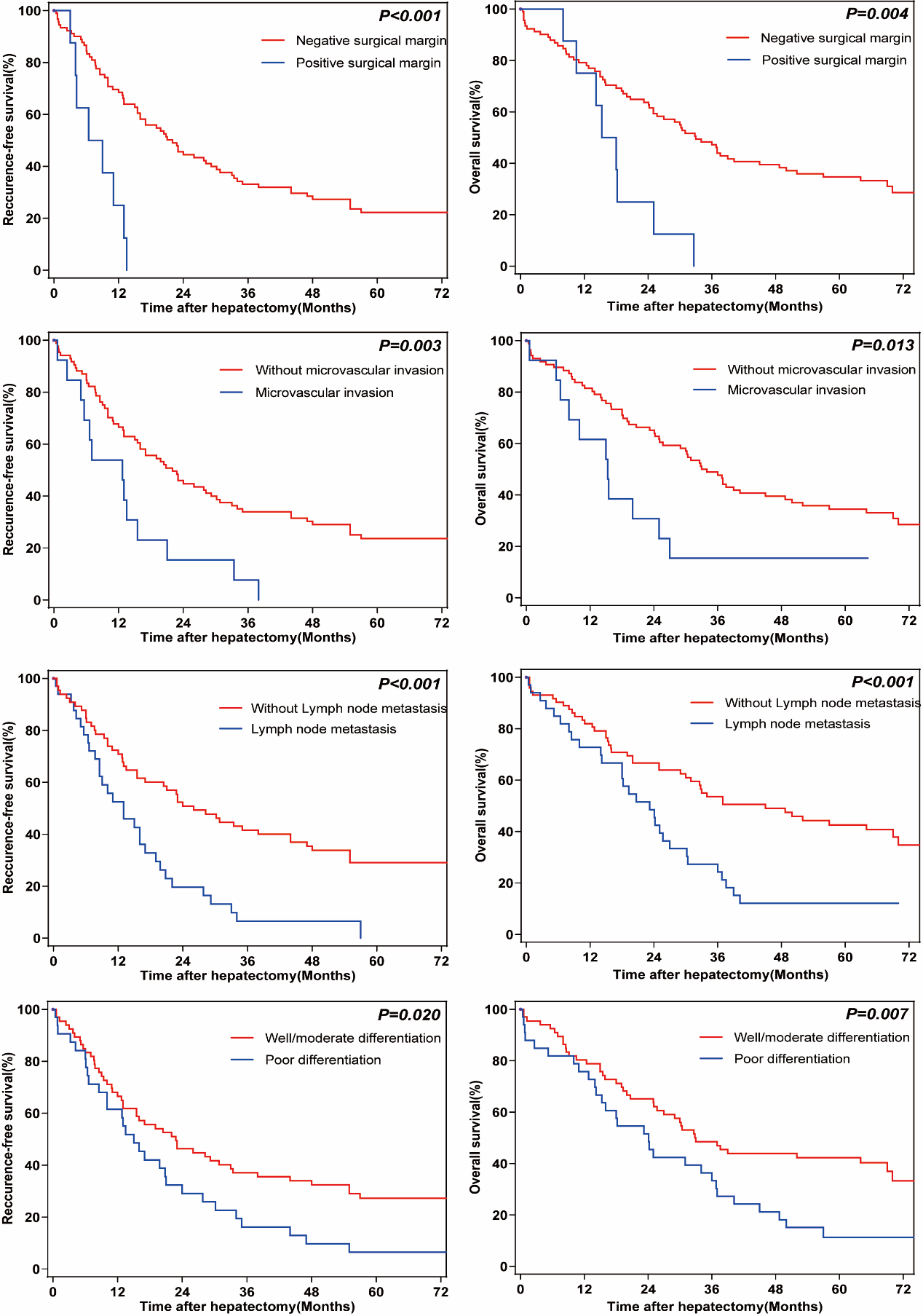


**Supplementary Figure S1.**  Recurrence-free survival (RFS) and overall survival (OS) in patients with different surgical margins, microvascular invasion, lymph node metastasis, and pathological differentiation.
